# Acute asymptomatic pulmonary thromboembolism detected using dynamic digital radiography following total hip arthroplasty: a case report

**DOI:** 10.1093/ehjcr/ytag427

**Published:** 2026-06-18

**Authors:** Yuta Hieda, Kazuma Miyatake, Hyonmin Choe, Koki Abe, Yutaka Inaba

**Affiliations:** Department of Orthopaedic Surgery, Yokohama City University, 3-9 Fukuura, Kanazawa-ku, Yokohama City, Kanagawa 236-0004, Japan; Department of Orthopaedic Surgery, Yokohama City University, 3-9 Fukuura, Kanazawa-ku, Yokohama City, Kanagawa 236-0004, Japan; Department of Orthopaedic Surgery, Yokohama City University, 3-9 Fukuura, Kanazawa-ku, Yokohama City, Kanagawa 236-0004, Japan; Department of Orthopaedic Surgery, Yokohama City University, 3-9 Fukuura, Kanazawa-ku, Yokohama City, Kanagawa 236-0004, Japan; Department of Orthopaedic Surgery, Yokohama City University, 3-9 Fukuura, Kanazawa-ku, Yokohama City, Kanagawa 236-0004, Japan

**Keywords:** Acute pulmonary thromboembolism, Total hip arthroplasty, Dynamic digital radiography, Case report

## Abstract

**Background:**

Acute pulmonary thromboembolism is a serious complication of total hip arthroplasty that may be asymptomatic, underscoring the importance of early detection. Contrast-enhanced computed tomography is the standard diagnostic modality; however, its use is limited by its invasiveness, radiation exposure, contrast allergy, and renal dysfunction. Dynamic digital radiography is a novel, minimally invasive imaging technique that enables visualization of pulmonary perfusion without contrast agents.

**Case summary:**

We report a case of a 58-year-old woman who underwent primary total hip arthroplasty. Post-operatively, the patient remained asymptomatic. One week after total hip arthroplasty, follow-up dynamic digital radiography demonstrated newly developed pulmonary perfusion abnormalities, with marked signal reduction in the right middle and lower lung zones, and a mild reduction in the left upper lung zone. Subsequent contrast-enhanced computed tomography confirmed bilateral pulmonary thromboembolism corresponding to perfusion defects detected using dynamic digital radiography. Anticoagulant therapy completely resolved the thromboembolism.

**Discussion:**

This case highlights the potential utility of dynamic digital radiography for early detection of asymptomatic acute pulmonary thromboembolism following total hip arthroplasty.

Learning points

- Asymptomatic pulmonary thromboembolism may occur after total hip arthroplasty and can be difficult to detect without imaging.
- Dynamic digital radiography is a low-invasive, contrast-free technique that may provide additional information on pulmonary perfusion.

## Introduction

Acute pulmonary thromboembolism is a serious complication of lower limb joint replacement surgery. Contrast-enhanced computed tomography (CT) is the preferred method for detecting embolism.^1,2^ However, some patients refuse CT because of concerns regarding radiation exposure, allergy to contrast agents, and renal dysfunction. In addition, asymptomatic pulmonary thromboembolism is not uncommon after surgery, and early detection remains challenging. Symptoms associated with pulmonary thromboembolism include dyspnoea and hypoxaemia in 0.14% of patients following total hip arthroplasty (THA) and 0.27% following total knee arthroplasty. Asymptomatic acute pulmonary thromboembolism was observed in 5% and 41% following THA and total knee arthroplasty, respectively.^3,4^ The current report describes a patient in whom dynamic digital radiography (DDR) imaging with a mobile radiography system (AeroDR TX m01; Konica Minolta, Inc., Tokyo, Japan) and a flat-panel detector (AeroDR fine motion; Konica Minolta, Inc., Tokyo, Japan) enabled the early detection of asymptomatic post-operative pulmonary thromboembolism with minimal invasiveness following the surgery.

## Summary figure

**Figure:**
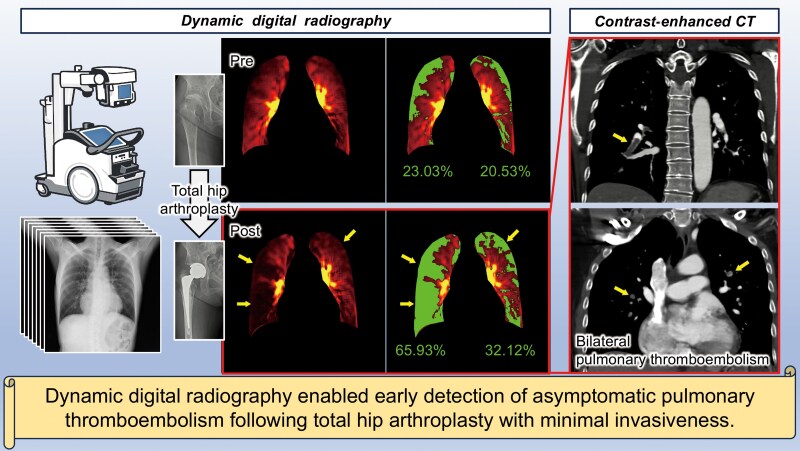


## Case presentation

A 58-year-old woman presented at our hospital for primary THA for secondary osteoarthritis, having undergone acetabular rotational osteotomy 25 years earlier for developmental dysplasia of the hip (*Figure 1*). Pre-operative physical findings included a height of 162 cm, weight of 47 kg, and body mass index of 17.9. Despite hip pain, she was able to walk independently with one free hand for up to 1 h. The blood test showed a D-dimer level of <500 ng/mL (reference < 700 ng/mL), and lower limb ultrasonography did not reveal deep vein thrombosis. Pre-operative DDR showed no abnormalities, and PH2-MODE, which visualizes pulmonary perfusion, demonstrated uniform signals in both lungs (*Figure 2*; Supplementary material online, *Movie S1*). Dynamic digital radiography was acquired in the supine position with a source-to-image distance of 120 cm using a tube voltage of 100 kV; an additional filter of 1 mm Al plus 0.2 mm Cu; a tube current of 32 mA; a pulse width of 5 ms; and a pulse rate of 15 frames per second over 7 s, yielding a total of 105 frames. PH2-MODE analysis was conducted using a dedicated X-ray motion analysis workstation (DI-X1; Konica Minolta, Inc., Tokyo, Japan).^5^ In PH2-MODE, the vertical lung field from the apex to the base was equally divided into three regions and separated into left and right sides, resulting in six regions in total, and motion correction was applied. Low-signal area mapping was applied to the PH2-MODE images. The low-signal area was defined as the proportion of the lung field occupied by regions with signal intensity below a predefined threshold.^6^ The threshold of 15 was set to the default value of the workstation, which was relative to the maximum intensity of a colour map; 15 was a reddish-black colour indicative of a perfusion defect. Previous studies have demonstrated that threshold-based analysis in DDR is typically optimized using receiver operating characteristic analysis rather than relying on a fixed universal value and that quantitative indices show excellent reproducibility (intraclass correlation coefficient > 0.9).^6^

**Figure 1:**
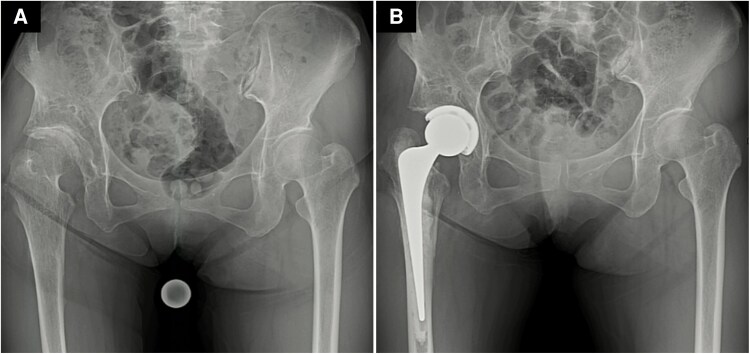
Pre- and post-operative hip X-ray images. (*A*) Pre-operative X-ray image of the front of the hip joint in supine position, showing osteoarthritis on right side. (*B*) Post-operative X-ray image after total hip arthroplasty using the cementless cup and cemented stem.

**Figure 2:**
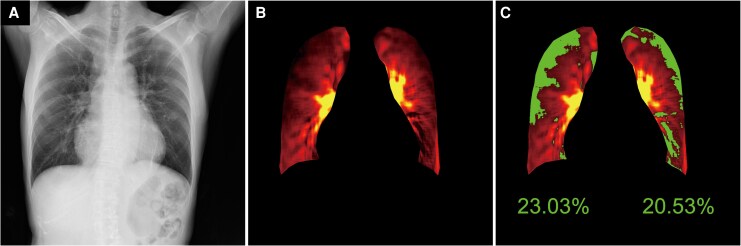
Pre-operative dynamic digital radiography images. (*A*) Chest radiograph (systolic frame extracted from the dynamic digital radiography video) showing no apparent abnormalities. (*B*) PH2-MODE image demonstrating no abnormal findings, with perfusion-related pulsation signals (red to yellow) uniformly distributed throughout the lung fields. (*C*) PH2-MODE with below-threshold, low-signal area mapping (threshold level = 15). Mild peripheral signal reduction is observed but does not show a wedge-shaped perfusion defect. The ratio of low-signal area (below threshold) to total lung area was 23.03% in the right lung and 20.53% in the left lung.

Unilateral THA was performed without intra-operative complications. The post-operative course was favourable, and she was able to transfer to a wheelchair the day after surgery. Mechanical thromboprophylaxis was used post-operatively, including a foot pump until the patient was able to walk, followed by elastic stockings. No pharmacological thromboprophylaxis was administered. The surgery was performed under general anaesthesia, and the operative time was 130 min. She began walking training with a walker 2 days after the surgery without dyspnoea, Homans’ sign, or pitting oedema. As part of our institutional prospective research protocol evaluating post-operative pulmonary perfusion using DDR, DDR was routinely performed on post-operative day 7 ± 2 regardless of symptoms. This protocol was approved by the institutional ethics committee, and the present case was part of a single-centre prospective study with a planned sample size of 444 cases. Post-operative imaging was conducted under the same conditions and with the same threshold as pre-operative imaging.

One week after surgery, DDR revealed perfusion abnormalities on PH2-MODE, with markedly decreased signals in the right, middle, and lower lung zones, which had not been present on pre-operative DDR. Mildly decreased signals were observed in the upper left lung zone. Blood testing indicated that the D-dimer level had increased to 9340 ng/mL, while troponin I was <4.7 pg/mL (reference < 26.2 pg/mL) and BNP was 27.9 pg/mL (reference < 18.4 pg/mL). Contrast-enhanced CT was conducted on the same day. Multiple filling defects were detected in both pulmonary arteries at the position corresponding to the signal decrease region in DDR (*Figure 3*; Supplementary material online, *Movie S2*). Deep vein thrombosis was detected in the soleus vein on the affected side using ultrasonography. Echocardiography showed pulmonary artery dilatation without pulmonary hypertension. The TAPSE was 19.2 mm, and RV TDI (S′/E′/A′) was 11.6/7.5/12.1 cm/s, suggesting no evidence of right ventricular dysfunction. Contrast-enhanced CT was performed before DDR interpretation, and DDR analysis was not used for real-time clinical decision-making. In our institution, post-operative contrast-enhanced CT is routinely performed as part of standard post-operative assessment, independent of DDR findings, D-dimer levels, or clinical probability scores.

**Figure 3:**
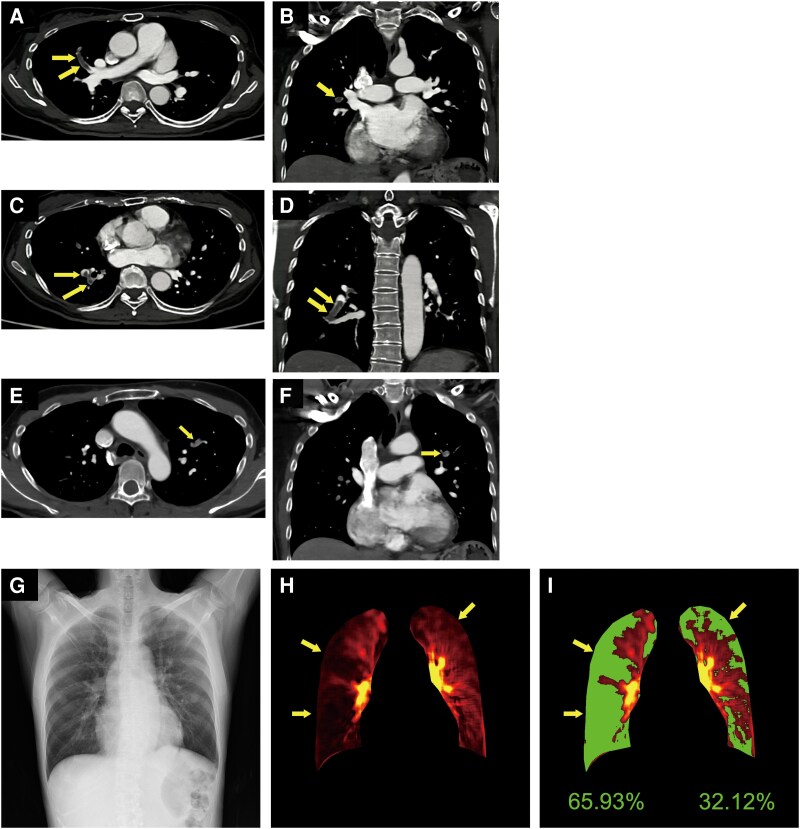
Contrast-enhanced computed tomography images 1 week after the surgery showing pulmonary thromboembolism. (*A* and *B*) Filling defects are observed in the right interlobar pulmonary artery extending to the right middle and lower lobar pulmonary arteries (yellow arrows). (*C* and *D*) Filling defects are observed in the right lower lobar pulmonary artery extending into the basal segmental branches (yellow arrows). (*E* and *F*) Filling defects are observed in the left upper lobar pulmonary artery (yellow arrows). (*G*) Chest radiograph (systolic frame extracted from the post-operative dynamic digital radiography video) showing no apparent abnormalities, similar to the pre-operative image. (*H*) PH2-MODE image showing decreased perfusion signals in the right middle and lower lung fields (yellow arrows), in contrast to the pre-operative PH2-MODE. (*I*) PH2-MODE with below-threshold low-signal area mapping using the same threshold level as the pre-operative image (*Figure 2*), clearly visualizing newly developed low-signal areas (below threshold) in the right middle and lower lung zones (yellow arrows). The ratio of low-signal area (below threshold) to total lung area increased to 65.93% in the right lung and 32.12% in the left lung.

Rivaroxaban 30 mg/day was administered for the first 3 weeks, followed by 15 mg/day for 3 months, and the patient remained without symptoms. After antithrombotic treatment, contrast-enhanced CT showed that the pulmonary thromboembolism had resolved (*Figure 4*). Lower limb ultrasonography showed resolution of the deep vein thrombosis, and echocardiography showed improvement in pulmonary artery dilatation with no indication of right ventricular overload.

**Figure 4:**
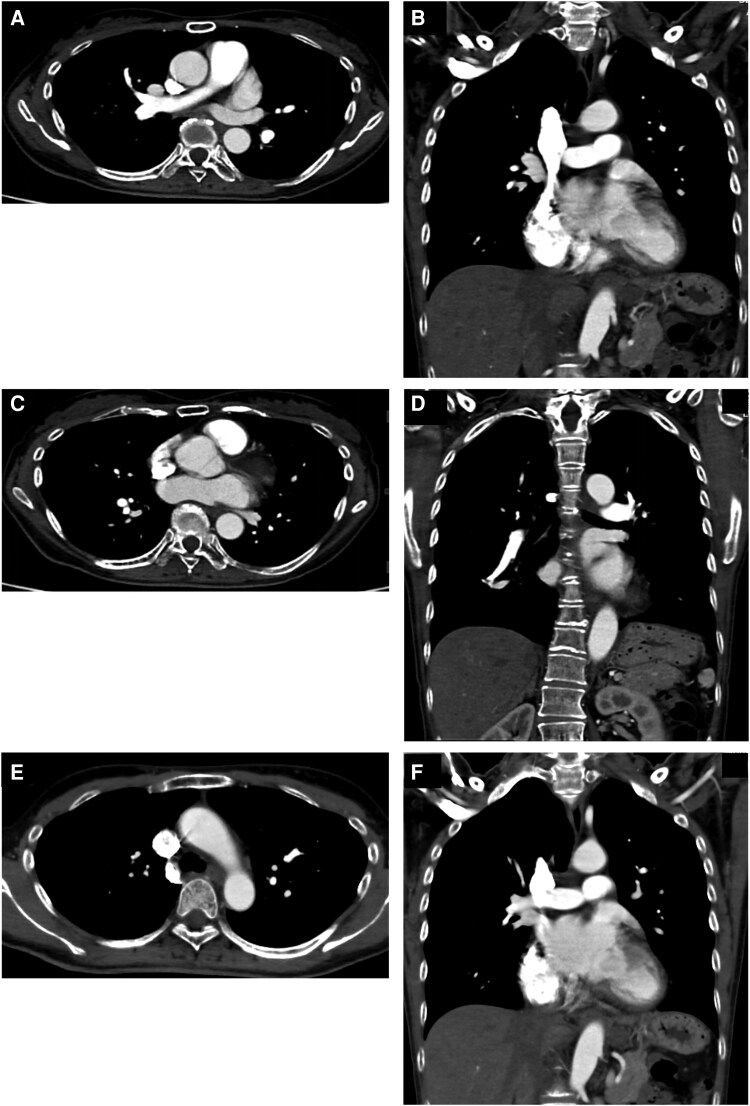
Contrast-enhanced computed tomography images 3 months after antithrombotic treatment. (*A* and *B*) Resolution of the filling defect in the right interlobar pulmonary artery. (*C* and *D*) Resolution of the filling defect in the right lower lobar pulmonary artery. (*E* and *F*) Resolution of the filling defect in the left upper lobar pulmonary artery.

## Discussion

Dynamic digital radiography is a novel, minimally invasive imaging method that enables rapid examination without the need for contrast agents or CT scans and without significant radiation exposure.^6–9^ Holding the breath for 7–10 s allows cardiac-related perfusion signals to be captured clearly, enabling areas of interrupted pulmonary blood flow caused by thromboembolism to appear as regions of low signal intensity, observed as well-defined wedge-shaped defects.^10,11^ This case suggests that DDR enables identification of asymptomatic pulmonary thromboembolism following THA.

Current guidelines recommend contrast-enhanced CT as the imaging modality of choice for suspected pulmonary thromboembolism.^3^ However, contrast-enhanced CT can be difficult in some cases because of patient concerns regarding contrast agent allergies, renal impairment, cost, lactic acidosis caused by biguanide diabetes medication, and radiation exposure. Dynamic digital radiography may provide complementary information in selected post-operative patients, with radiation exposure comparable to that of standard chest X-ray imaging. The corresponding effective dose of DDR (0.2 mSv) was substantially lower than that of a lung ventilation–perfusion (V/Q) scan (2 mSv) or contrast-enhanced CT (3–6 mSv).^12–15^ Adding DDR to conventional chest radiography provided high diagnostic accuracy for pulmonary thromboembolism, with sensitivity and specificity of 0.70 and 0.93 (standing) and 0.66 and 0.92 (supine), respectively.^13^ Dynamic digital radiography may be positioned as a simple adjunctive option for pulmonary perfusion assessment. Research is required to demonstrate sensitivity and achieve standardization of acquisition and analysis parameters (position, frame rate, thresholding, and segmentation).

Limitations of the DDR include the potential for errors in heart rhythm and blood flow assessment due to patient movement during the test, difficulty in visualizing blood flow in the left lower lobe obscured by the heart on radiography, and the possibility that patients are unable to hold their breath. In addition, this is a single-case report, which limits the generalizability of the findings. The analysis was not performed in a blinded manner, and quantitative validation against established perfusion imaging modalities was not conducted. Furthermore, long-term follow-up, including the evaluation of chronic thromboembolic pulmonary hypertension, was not available in this case. The clinical benefit of early treatment for asymptomatic pulmonary embolism after the surgery has not been well established. Antithrombotic therapy for asymptomatic pulmonary embolism may increase post-operative haematoma size and potentially cause post-operative infection. Overtriage of asymptomatic pulmonary thromboembolism for DDR and unnecessary treatment requires caution; obtaining full informed consent from the patient is essential before proceeding with treatment. The role of DDR as a screening tool should be evaluated in future prospective studies.

## Conclusion

This case report describes a patient in whom asymptomatic acute pulmonary thromboembolism following THA was detected using DDR. Dynamic digital radiography may be a useful adjunctive imaging modality for the evaluation of post-operative pulmonary thromboembolism.

## Supplementary Material

ytag427_Supplementary_Data

## Data Availability

All the data supporting our findings are contained within the manuscript.

## Lead author biography

Kazuma Miyatake, MD, PhD, is an orthopaedic surgeon at Yokohama City University Hospital. He graduated from Yokohama City University in 2011. He joined the Department of Orthopaedic Surgery, Yokohama City University Graduate School, in 2016. He became an attending physician in 2020, earned his PhD in 2021, and has served as an assistant professor since 2021.

## Notes

**Consent:** The authors confirm that written consent for submission and publication of this case report including images and associated text has been obtained from the patient, in line with COPE guidance.
